# Neurovascular Relationships in AGEs-Based Models of Proliferative Diabetic Retinopathy

**DOI:** 10.3390/bioengineering11010063

**Published:** 2024-01-08

**Authors:** Juan S. Peña, Ranjini K. Ramanujam, Rebecca A. Risman, Valerie Tutwiler, Francois Berthiaume, Maribel Vazquez

**Affiliations:** Department of Biomedical Engineering, Rutgers, The State University of New Jersey, Piscataway, NJ 08854, USA

**Keywords:** advanced glycation end products, diabetic retinopathy, vascular disease, RAGE, inner blood retinal barrier

## Abstract

Diabetic retinopathy affects more than 100 million people worldwide and is projected to increase by 50% within 20 years. Increased blood glucose leads to the formation of advanced glycation end products (AGEs), which cause cellular and molecular dysfunction across neurovascular systems. These molecules initiate the slow breakdown of the retinal vasculature and the inner blood retinal barrier (iBRB), resulting in ischemia and abnormal angiogenesis. This project examined the impact of AGEs in altering the morphology of healthy cells that comprise the iBRB, as well as the effects of AGEs on thrombi formation, in vitro. Our results illustrate that AGEs significantly alter cellular areas and increase the formation of blood clots via elevated levels of tissue factor. Likewise, AGEs upregulate the expression of cell receptors (RAGE) on both endothelial and glial cells, a hallmark biomarker of inflammation in diabetic cells. Examining the effects of AGEs stimulation on cellular functions that work to diminish iBRB integrity will greatly help to advance therapies that target vision loss in adults.

## 1. Introduction

Diabetes mellitus is a growing metabolic disorder that impacts a staggering 10% of the global population, with medical costs over USD $500 billion per year [1]. Over one third of adult diabetics will suffer vision loss through diabetic retinopathy, where chronically elevated blood glucose, or hyperglycemia, causes abnormal blood vessel growth inside and around layers of photosensitive retinal neurons. Chronic and uncontrolled hyperglycemia is also known to increase the production of advanced glycation end products (AGEs), which are harmful pro-inflammatory compounds that irreversibly change protein structure/function and are strongly implicated in neurodegeneration [2]. AGEs can naturally form in the blood as a result of non-enzymatic glycosylation of proteins and lipids vastly found in contemporary diets. 

**Figure 1:**
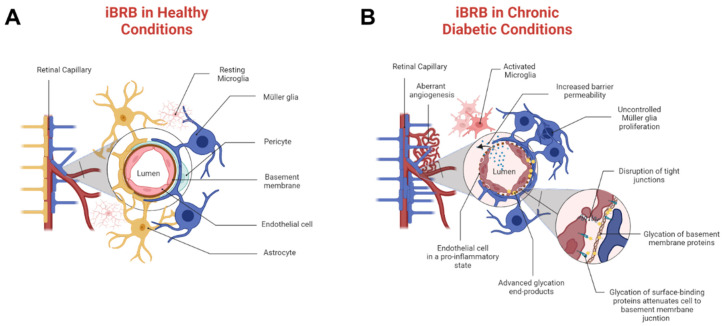
Schematic of the inner blood–retinal barrier (iBRB) that regulates the flux of nutrients and oxygen between circulating blood and the neural retina. (**A**) Healthy iBRB comprises endothelial cells, pericytes, astrocytes, and Müller glia. (**B**) Pathogenic iBRB exhibits aberrant angiogenesis and chronic advanced glycation end products (AGEs) that cause loss of pericytes and astrocytes. The detrimental effects of AGEs in cell communication, vessel structure, and pro-inflammation are shown in the circular inset. Communication across the barrier tissue can be strongly regulated by surviving endothelial cells and Müller glia.

Diabetic retinopathy is a progressive disease that becomes proliferative diabetic retinopathy (PDR) in its most severe stage and produces alarming health disparities in the vision loss of mature adults [3]. Retinal hemorrhage and thrombosis are a consequence of diabetes and a hallmark of progression to PDR [4]. Hyperglycemia and AGEs stimulate abnormal angiogenesis that primarily disrupts the inner blood–retinal barrier (iBRB), a selective neurovascular tissue that regulates the transport of molecules across microcapillaries and the neural retina. A healthy iBRB comprises endothelial cells, pericytes, Müller glia, and astrocytes, as shown in Figure 1A. By contrast, pathological tissue is represented by Figure 1B, where chronic environments of AGEs cause overwhelming pericyte death, activation of microglia, and severe astrogliosis [5]. As shown, molecular transport in PDR can be strongly regulated through endothelial cell and Müller glia communication, whose cell to cell signaling remains vastly understudied. Moreover, pathogenic iBRB is likely further compromised by the presence of retinal thrombi [6] that can impact both barrier integrity and molecular transport across it. The influences of chronic AGEs on pathological responses are incompletely understood, despite the potential to advance the development of therapies for adult tissue.

This project examined relative relationships between endothelial cells and the two retinal neuroglia cell types, Müller glia and astrocytes, in chronic environments of high glucose and AGEs used to model PDR, in vitro. Experiments studied phenotypic changes across cells when cultured in the conditioned medium of neighboring cells as well as relative expression of the receptor for AGEs, called RAGE. Results illustrate that Müller glia respond to endothelial cells with significant changes in cell area, which dramatically increases when cultured within AGEs medium. RAGE expression was also seen to increase by similar amounts in both cell types upon AGEs stimulation. Further, AGEs were shown to increase tissue factor (TF), a primary cellular initiator of blood coagulation known to initiate the clotting cascade [6]. Biotechnologies to evaluate and mitigate the impacts of AGEs across pathogenic iBRB will greatly improve treatments for neurovascular eye diseases and reduce adult vision loss. 

## 2. Materials and Methods

### 2.1. Cell Culture

Primary Müller glia were isolated from the retina of adult female wild-type Sprague-Dawley rats using a Papain dissociation kit (Worthington, Lakewood, NJ, USA), as per established protocols [7]. Primary rat retinal endothelial cells were commercially purchased (CellBiologics, RA6065, Chicago, IL, USA) as were rat retinal astrocytes (ScienCell, R1870, Carlsbad, CA, USA). All cells were cultured in T-75 polystyrene flasks with 88% 5 mM low glucose (ThermoFisher, 12320032, Carlsbad, CA, USA), 10% fetal bovine serum (FBS) (ThermoFisher, 26140, Carlsbad, CA, USA), and 2% penicillin/streptomycin (VWR, K952, Philadelphia, PA, USA). Astrocytes and Müller glia used Dulbecco’s modified Eagle medium (ThermoFisher, 12320032, Carlsbad, CA, USA) as the base medium, while endothelial cells were grown in endothelial complete medium (CellBiologics, M1266, Chicago, IL, USA), supplemented with 2% FBS, 0.1% epidermal growth factor, 0.1% vascular endothelial growth factor, and 1% antibiotic/antimycotic solution. Cells exposed to AGEs were cultured in high-glucose medium (25 mM) (VWR, 76470-182, Philadelphia, PA, USA) with advanced glycation end products (AGEs) (MilliPore Sigma, 121800-M, St. Louis, MO, USA) at a concentration of 0.01 mg/mL for 15 days prior to performing any study. Cell cultures were maintained in a tissue culture incubator at 5% CO_2_ and 37 °C, and media were replaced every 2–3 days. 

### 2.2. Conditioned Media

Endothelial cells were cultured in 24-well plates at a concentration of 250,000 cells/mL in basal low-glucose DMEM for 3 days. Tests collected the medium from endothelial cells and filtered the solutions through 0.2 µm pore filters prior to testing, as per the literature [8].

### 2.3. AGEs/RAGE

Advanced glycation end products (AGEs) were purchased (MilliPore Sigma, 121800-M, St. Louis, MO, USA) and used at a concentration of 0.01 mg/mL in high-glucose medium (25 mM) (VWR, 76470-182, Philadelphia, PA, USA) for all tests. The RAGE receptor was imaged using immunocytochemistry (ICC). Primary antibody (ThermoFisher, PA524787, Carlsbad, CA, USA) and secondary antibody (ThermoFisher, R37118, Carlsbad, CA, USA).

### 2.4. Cell Morphology

Cell morphology was evaluated based on changes in cell area over time. Cells seeded in 24-well plates at a concentration of 250,000 cells/mL were imaged at 6 h, 12 h, 24 h, and 48 h post-seeding. ImageJ was used to quantify the total area occupied by the cells at respective timepoints.

### 2.5. Immunocytochemistry

Briefly, cells were seeded in 24-well plates (VWR, 29442-044, Philadelphia, PA, USA) at a concentration of 2.5 × 105 cells/mL and allowed to attach for 24 h. Media from each well were removed, and wells were washed 3 times with Dulbecco’s phosphate-buffered saline (DPBS) (Sigma-Aldrich, Cat No. D8537, Allentown, PA, USA), and cells were fixed with cold paraformaldehyde (4%) for 5 min. Then, wells were washed with DPBS for 5 min twice at room temperature. Blocking buffer solution (0.05% Triton X-100, 2% donkey serum, and 3% BSA in DPBS) was added to each well for 15 min at room temperature. Following that, wells were washed twice with DPBS for 2 min, then a primary antibody for the receptor of Advanced glycation end products (ThermoFisher, PA524787, Carlsbad, CA, USA) was added to each well and incubated overnight. The next day, each well was washed 3 times with DPBS for 2 min, followed by the addition of the secondary antibody solution (ThermoFisher, R37118, Carlsbad, CA, USA) for 1 h at room temperature. Wells were washed with DPBS for 2 min 3 times, before adding DAPI (1:1000) (ThermoFisher, D1306, Carlsbad, CA, USA) into each well for 5 min at room temperature. Each well was washed with DPBS 3 times for 2 min. Receptor expression was evaluated via fluorescence microscopy (Leica Microsystems, DMi8, Chicago, IL, USA). 

### 2.6. Turbidity

Commercially available human-pooled plasma (Cone Bioproducts, 5781, Seguin, TX, USA) starting fibrinogen concentration 2.9 mg/mL) was warmed to 37 °C before all experiments. Clots for turbidity assays were formed with plasma, 25 mM CaCl_2_, and tissue factor (TF) at their respective concentrations (30, 75, and 600 pM). Then, 100 μL of plasma mix was added to 96-well plates and transferred to a SpectraMax plate reader. The optical density was recorded until a plateau was reached at a wavelength of 405 nm and a temperature of 37 °C. Data were normalized to the first point. Rate of formation was measured as the slope of the linear region. At least three replicates per condition were tested.

### 2.7. Imaging Analysis

An inverted epifluorescence microscope (Leica DMi8) was used to observe cell behavior over time and to perform optical analyses with a cooled CCD camera (Leica Microsystems, DFC7000 GT, Chicago, IL, USA) via a 10× objective. Images were evaluated using ImageJ with 12-bit data, as carried out previously by our group [9].

To perform scanning electron microscopy (SEM) (Hitachi S-4800 FE-STEM-EDS with 20 kV at 10 k×), clots were performed with the conditions reflected in turbidity in etched Delrin molds. All samples were washed with sodium cacodylate for 1 h, fixed with 2% glutaraldehyde overnight, and washed again with 50 mM cacodylate buffer containing 150 mM NaCl (pH 7.4). Clots were dehydrated with increasing concentrations of ethanol (30–100% *v*/*v*) and hexamethyldisilazane. Samples were sputter coated with 10 nm of gold/palladium (Quorum Technologies, EMS 150T ES, Sacramento, CA, USA). 

### 2.8. Statistical Analysis

All experiments were performed with a minimum of n = 3 (triplicate); for cell experiments, each used approximately 50 cells per condition. Normal data were evaluated using a one-way-ANOVA test, with a Tukey post-hoc test. Symbols: ** = *p* < 0.01, *** = *p* < 0.01, **** = *p* < 0.001.

## 3. Results and Discussion

**Figure 2:**
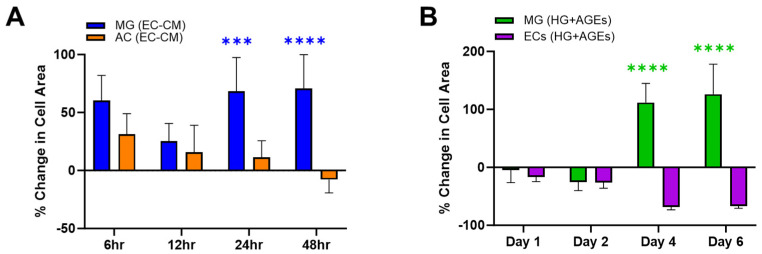
The composition of different extracellular media produces changes in the average area of endothelial cells (ECs), astrocytes (AC), and Müller glia (MG). (**A**) The percentage change in average area of MG becomes larger in response to endothelial cell-conditioned medium (EC-CM). While the area of AC transiently increases and then returns to basal levels. Percentage change of cell area was normalized to each cell type control. (**B**) Medium of high glucose (HG: 25 mM) and advanced glycation end products (AGEs) cause decreases in average area of endothelial cells (n = 20) and increases in Müller glia (n = 20) cell area. Significance shown as *** *p* < 0.001 and **** *p* < 0.0001.

The experiments first measured changes in adhered surface area when cells were exposed to the secretome of other cells in their medium. Figure 2A illustrates the percentage change in surface area for Müller glia (MG) and astrocytes (AC) when cultured in endothelial cell (EC)-conditioned medium. MG were seen to increase in surface area by up to 50% when cultured in EC-conditioned medium. By contrast, AC responded to EC-conditioned medium with a decreasing cell area, which became smaller than the control over the experimental time period. These different phenotypical changes of retinal glia, in vitro, are consistent with their respective functions, in vivo. MG span the neural retina to provide trophic support between retinal neurons, regulate uptake of neurotransmitters, control the volume and water content of the retinal extracellular space, and remove waste to maintain the pH (rev in [10,11]). Significantly, MG also regulate the redox signaling needed for phototransduction, which leads to the exceptionally high metabolic rate of retina and its elevated consumption of glucose. In this role, MG manage the production of reactive oxygen species that can damage cell structures and work intimately with the retinal vasculature to safeguard the retinal supply of oxygen and nutrients [9,12]. In complement, AC are the predominant neuroglia of the blood–brain barrier (BBB), where the cells control the ionic and water balance, as well as regulate the composition of extracellular brain fluid, interactions with immune cells, and synaptic functions (Rev in [13]). However, while MG reside within the neural retina, AC cell bodies are located upon the nerve fiber layer of healthy retina and extend only their cell processes within the neural retina [13]. Surprisingly, many barrier models include only AC to study the role of glia in different neurovascular barriers [14,15]. However, PDR patients often undergo vitrectomy to remove scarred tissue made of reactive astrocytes upon the nerve fiber layer [16], leaving pathogenic iBRB with strongly diminished numbers of AC and, consequently, stronger reliance on interactions between MG and EC. These differences in glial phenotypic observations in vitro echo significant differences in AC and MG functions in healthy and degenerated retina. Further studies will identify the cytokine composition of conditioned media to establish a more direct relationship between cell area changes and cytokine expression. The data highlight the benefits of controlled in vitro study to aid the development of therapies by examining cell–cell relationships in chronic pathogenic states.

**Figure 3:**
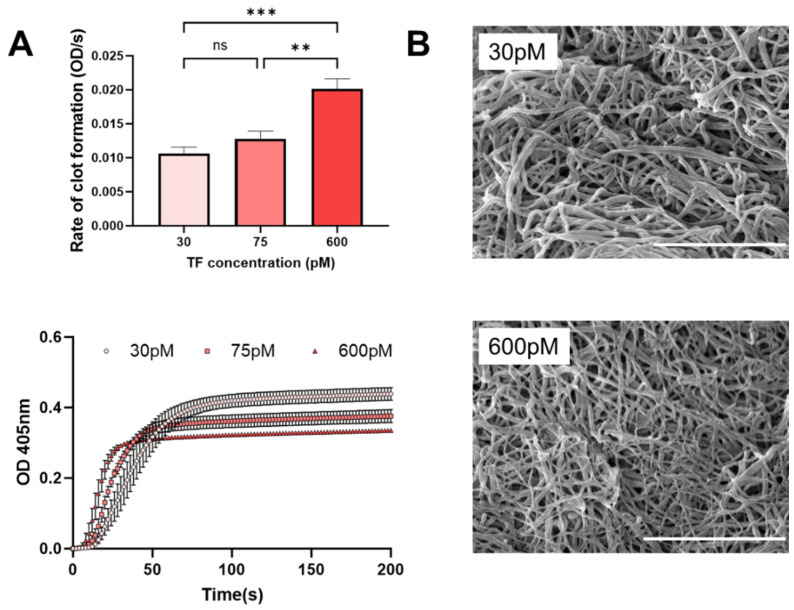
Tissue factor (TF) concentration generates changes in clotting kinetics and in clot structure. (**A**) Increasing TF concentration yields a faster rate of clot formation in human donor blood plasma as determined with turbidity measurements over time. (**B**) Altered structure of blood clots is visualized with scanning electron microscopy (SEM) images (Scale bar = 5 μm). Significance shown as ** *p* < 0.01 and *** *p* < 0.001, ns = no significance.

Experiments next examined cell changes in EC and MG within microenvironments of high glucose and AGEs stimulation, known to be a hallmark of PDR. Figure 2B illustrates substantial increases in MG surface area over 6 days compared to its control (medium only). By contrast, EC decreases in size within AGEs environments, which is consistent with the in vivo literature [6]. ECs line the retinal vasculature and rely upon junctional complexes for selective permeability of molecules via para- and trans-cellular pathways [17]. Decreases in EC size have been shown to diminish cell connectivity and increase barrier permeability [18]. Further, RAGE expression leads to altered F-actin organization and impaired membrane re-sealing in EC that decreases cell size and increases transcellular transport [19]. These responses are significant to PDR because tight junctions between EC produce surfaces that discourage the attachment of cells and associated clotting proteins to prevent thrombosis. The AGEs-decreased size of EC observed in vitro may point to increased potential for the formation of blood clots, which are the primary clinical indicators of PDR^2^. Moreover, diabetic patients are prothrombotic with an increased risk of clot formation linked to the excessive generation of tissue factor (TF) [6], which initiates the clotting cascade. In particular, AGEs bind to transmembrane RAGE proteins and activate endothelial cells, monocytes, and/or macrophages to over synthesize TF [20,21,22]. Figure 3 illustrates that increasing TF yields an accelerated rate of clot formation and altered clot structures. A decrease in fiber diameter (137 to 64 nm, *p* < 0.0001) was observed when TF concentration was increased from 30 to 600 pM (Figure 3B). Thus, AGEs may have a dual effect on iBRB integrity via clotting processes, where retinal thrombosis offers an additional therapeutic target for modulating transport in PDR. 

**Figure 4:**
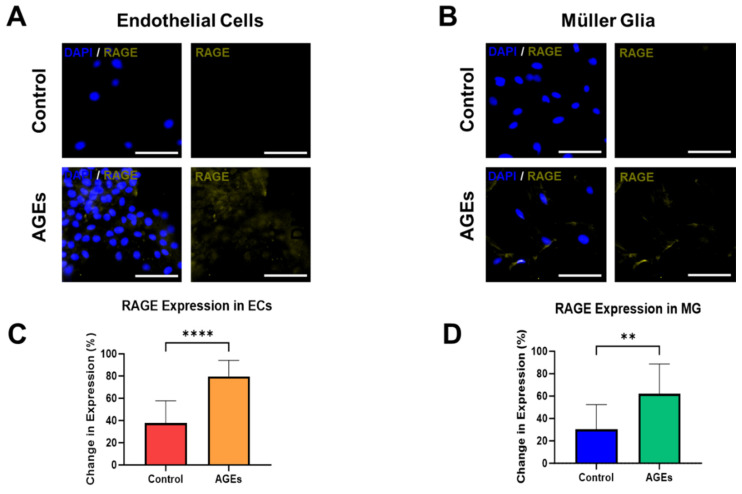
Stimulation with medium containing high glucose (25 mM) and advanced glycation end products (AGEs) results in altered expression of the transmembrane receptor, RAGE, in endothelial cells and Müller glia. Representative images of (**A**) endothelial cells stained for the nucleus (blue) and RAGE (orange), and (**B**) Müller glia against controls (respective mediums only). Measured differences of average fluorescence expression per cell were normalized to each control (**C**) endothelial cells and (**D**) Müller glia (Scale bar = 100 μm). Significance shown as ** *p* < 0.01 and **** *p* < 0.0001.

The final experiments measured the expression of RAGE, the receptor for AGEs, within EC and MG, in both the control medium and medium spiked with AGEs and high glucose. Rising levels of AGEs are significant biomedical markers that change very slowly in vivo [2]. These dynamics render in vitro systems as highly suitable platforms to study the effects of AGEs and model chronic conditions of PDR over time. Figure 4A depicts representative fluorescent images that illustrate minimal RAGE expression upon EC cultured in control medium, but significantly increased expression in EC cultured with AGEs. Interestingly, EC cultured with AGEs seemed to cluster, while EC in the control medium did not. The changes are denoted by differences in the average fluorescence intensity per cell, which increase by over 50% to illustrate significant changes (*p* < 0.01), as quantified in Figure 4C. Similarly, MG cultured in the control medium expressed lower RAGE expression than when cultured in AGEs conditions, with significant changes in average fluorescence intensity per cell that increase by over 33% (*p* < 0.01), as shown in Figure 4B,D. RAGE expression in the control and AGEs groups of endothelial cells and Muller glia cells was ubiquitously expressed in the cell cytoplasm. The seemingly higher cell density in the endothelial cell group exposed to AGEs is a result of cell clustering in this group. The majority of cells in the endothelial cell group exposed to AGEs tended to cluster, which was not observed in the control group. Concurrent RAGE activation on EC and MG is especially significant in PDR because the binding activates signaling to promote intracellular generation of reactive oxygen species (ROS), activation of the pro-inflammatory transcription factor NF-κB, and expression of multiple inflammation-related markers [23,24]. Further, RAGE activation of EC causes overexpression of RAGE in a feedforward loop that leads to the expression of intercellular adhesion molecule-1 (ICAM) and a disruption of tight junctions needed to maintain iBRB integrity [25]. Similarly, RAGE activation on MG cells leads to the production of pro-inflammatory cytokines and elevated vascular endothelial growth factor (VEGF) [26], both of which can diffuse to nearby EC and add to RAGE-mediated signals that promote disruption of the iBRB. Furthermore, future studies can apply Western blot assays, high-performance liquid chromatography, and/or mass spectrometry to measure quantitative levels of RAGE expression in diabetic cells. These data underscore the need for detailed examination of AGEs impacts on neurovascular communication in pathogenic barrier tissues.

## 4. Conclusions

The integrity of pathogenic blood–retinal barriers relies upon complex cell to cell communication between endothelial cells and Müller glia, whose impact from chronic AGEs is underexplored. Cognate relationships between these two cell types are becoming increasingly elucidated through evidence of significant Müller glia functions in retinal regeneration and repair [10], as well as newfound roles of endothelial cells in blood clotting. Studies to examine the effects of AGEs on the changing integrity and altered transport mechanisms across pathogenic barriers of PDR would greatly advance the development of novel treatments to prevent vision loss in adults. 

## Data Availability

The data presented in this study are available on request from the corresponding author.

## References

[1] Teo Z.L., Tham Y.C., Yu M., Chee M.L., Rim T.H., Cheung N., Bikbov M.M., Wang Y.X., Tang Y., Lu Y. (2021). Global Prevalence of Diabetic Retinopathy and Projection of Burden through 2045: Systematic Review and Meta-analysis. Ophthalmology.

[2] Xu J., Chen L.-J., Yu J., Wang H.-J., Zhang F., Liu Q., Wu J. (2018). Involvement of Advanced Glycation End Products in the Pathogenesis of Diabetic Retinopathy. Cell. Physiol. Biochem..

[3] Peña J.S., Vazquez M. (2019). Reducing health disparities in adult vision loss via interfaces with emerging technology. Eye.

[4] Xiao H., Tang J., Zhang F., Liu L., Zhou J., Chen M., Li M., Wu X., Nie Y., Duan J. (2023). Global trends and performances in diabetic retinopathy studies: A bibliometric analysis. Front. Public Health.

[5] Ferland-McCollough D., Slater S., Richard J., Reni C., Mangialardi G. (2017). Pericytes, an overlooked player in vascular pathobiology. Pharmacol. Ther..

[6] Picard F., Adjedj J., Varenne O. (2017). Diabetes Mellitus, a prothrombotic disease. Ann. Cardiol. Angeiol..

[7] Pereiro X., Ruzafa N., Acera A., Urcola A., Vecino E. (2020). Optimization of a Method to Isolate and Culture Adult Porcine, Rats and Mice Muller Glia in Order to Study Retinal Diseases. Front. Cell. Neurosci..

[8] Dowling P., Clynes M. (2011). Conditioned media from cell lines: A complementary model to clinical specimens for the discovery of disease-specific biomarkers. Proteomics.

[9] Cliver R.N., Castro N., Russomano T., Lardieri G., Quarrie L., van der Merwe H., Vazquez M. (2022). Antioxidants derived from natural products reduce radiative damage in cultured retinal glia to prevent oxidative stress. Neuroglia.

[10] Peña J.S., Vazquez M. (2022). Harnessing the Neuroprotective Behaviors of Muller Glia for Retinal Repair. Front. Biosci. Landmark Ed..

[11] Reichenbach A., Bringmann A. (2020). Glia of the human retina. Glia.

[12] Albert-Garay J.S., Riesgo-Escovar J.R., Salceda R. (2022). High glucose concentrations induce oxidative stress by inhibiting Nrf2 expression in rat Muller retinal cells in vitro. Sci. Rep..

[13] Díaz-Castro B., Robel S., Mishra A. (2023). Astrocyte Endfeet in Brain Function and Pathology: Open Questions. Annu. Rev. Neurosci..

[14] Bora K., Kushwah N., Maurya M., Pavlovich M.C., Wang Z., Chen J. (2023). Assessment of Inner Blood-Retinal Barrier: Animal Models and Methods. Cells.

[15] Maurissen T.L., Pavlou G., Bichsel C., Villaseñor R., Kamm R.D., Ragelle H. (2022). Microphysiological Neurovascular Barriers to Model the Inner Retinal Microvasculature. J. Pers. Med..

[16] Chang W.C., Lin C., Lee C.H., Sung T.L., Tung T.H., Liu J.H. (2017). Vitrectomy with or without internal limiting membrane peeling for idiopathic epiretinal membrane: A meta-analysis. PLoS ONE.

[17] Hosoya K., Tachikawa M. (2012). The inner blood-retinal barrier: Molecular structure and transport biology. Adv. Exp. Med. Biol..

[18] Robles-Osorio M.L., Sabath E. (2023). Tight junction disruption and the pathogenesis of the chronic complications of diabetes mellitus: A narrative review. World J. Diabetes.

[19] Xiong F., Leonov S., Howard A.C., Xiong S., Zhang B., Mei L., McNeil P., Simon S., Xiong W.C. (2011). Receptor for advanced glycation end products (RAGE) prevents endothelial cell membrane resealing and regulates F-actin remodeling in a beta-catenin-dependent manner. J. Biol. Chem..

[20] Soma P., Swanepoel A.C., Bester J., Pretorius E. (2017). Tissue factor levels in type 2 diabetes mellitus. Inflamm. Res..

[21] Ichikawa K., Yoshinari M., Iwase M., Wakisaka M., Doi Y., Iino K., Yamamoto M., Fujishima M. (1998). Advanced glycosylation end products induced tissue factor expression in human monocyte-like U937 cells and increased tissue factor expression in monocytes from diabetic patients. Atherosclerosis.

[22] Stirban A., Gawlowski T., Roden M. (2014). Vascular effects of advanced glycation endproducts: Clinical effects and molecular mechanisms. Mol. Metab..

[23] Kaur G., Singh N.K. (2021). The Role of Inflammation in Retinal Neurodegeneration and Degenerative Diseases. Int. J. Mol. Sci..

[24] Olekson M.P., Faulknor R.A., Hsia H.C., Schmidt A.M., Berthiaume F. (2016). Soluble Receptor for Advanced Glycation End Products Improves Stromal Cell-Derived Factor-1 Activity in Model Diabetic Environments. Adv. Wound Care.

[25] Nonaka K., Kajiura Y., Bando M., Sakamoto E., Inagaki Y., Lew J.H., Naruishi K., Ikuta T., Yoshida K., Kobayashi T. (2018). Advanced glycation end-products increase IL-6 and ICAM-1 expression via RAGE, MAPK and NF-kappaB pathways in human gingival fibroblasts. J. Periodontal Res..

[26] Zong H., Ward M., Madden A., Yong P.H., Limb G.A., Curtis T.M., Stitt A.W. (2010). Hyperglycaemia-induced pro-inflammatory responses by retinal Muller glia are regulated by the receptor for advanced glycation end-products (RAGE). Diabetologia.

